# C-Reactive Protein Causes Adult-Onset Obesity Through Chronic Inflammatory Mechanism

**DOI:** 10.3389/fcell.2020.00018

**Published:** 2020-02-20

**Authors:** Qiling Li, Qi Wang, Wei Xu, Yamin Ma, Qing Wang, Danita Eatman, Shaojin You, Jin Zou, James Champion, Lanbo Zhao, Ye Cui, Wenzhi Li, Yangyang Deng, Li Ma, Biao Wu, Guangdi Wang, Xiaodong Zhang, Qingwei Wang, Mohamed A. Bayorh, Qing Song

**Affiliations:** ^1^Department of Obstetrics and Gynecology, First Affiliated Hospital, Xi'an Jiaotong University, Xi'an, China; ^2^Cardiovascular Research Institute and Department of Medicine, Morehouse School of Medicine, Atlanta, GA, United States; ^3^Department of Pharmacology & Toxicology, Morehouse School of Medicine, Atlanta, GA, United States; ^4^Histo-Pathology Core, Atlanta Research & Educational Foundation, Atlanta VA Medical Center, Decatur, GA, United States; ^5^Center for Cancer Research and Therapeutic Development, Clark Atlanta University, Atlanta, GA, United States; ^6^Center for Laboratory Animal Resources, Morehouse School of Medicine, Atlanta, GA, United States; ^7^Department of Mathematics and Statistics, Georgia State University, Atlanta, GA, United States; ^8^Institute of Artificial Intelligence and Robotics, Xi'an Jiaotong University, Xi'an, China; ^9^Key Laboratory of Synthetic and Natural Functional Molecule Chemistry of the Ministry of Education, College of Chemistry and Materials Science, Northwest University, Xi'an, China; ^10^Department of Chemistry, Xavier University of Louisiana, New Orleans, LA, United States; ^11^Yerkes Imaging Center MRI Core, Yerkes National Primate Research Center, Emory University, Atlanta, GA, United States

**Keywords:** CRP, obesity, inflammation, gut flora, adult-onset

## Abstract

Obesity is characterized by low-grade chronic inflammation. As an acute-phase reactant to inflammation and infection, C-reactive protein (CRP) has been found to be the strongest factor associated with obesity. Here we show that chronic elevation of human CRP at baseline level causes the obesity. The obesity phenotype is confirmed by whole-body magnetic resonance imaging (MRI), in which the total fat mass is 6- to 9- fold higher in the CRP rats than the control rats. Univariate linear regression analysis showed different growth rates between the CRP rats and the control rats, and that the difference appears around 11 weeks old, indicating that they developed adult-onset obesity. We also found that chronic elevation of CRP can prime molecular changes broadly in the innate immune system, energy expenditure systems, thyroid hormones, apolipoproteins, and gut flora. Our data established a causal role of CRP elevation in the development of adult-onset obesity.

## Introduction

Chronic inflammation is a player in the progressive process of obesity development (Christ et al., 2018; Piening et al., 2018). In a recent human longitudinal weight perturbation study, researchers noticed that even modest weight gain was associated with inflammatory activation (Piening et al., 2018). As a signature marker for systemic inflammations, C-reactive protein (CRP) has been consistently shown to be the strongest factor associated with overweight and obesity in human epidemiological studies (Timpson et al., 2011) and was thought to be the consequence of obesity rather than the cause of obesity by a statistical approach (Timpson et al., 2011).

On the other hand, accumulating evidences suggest that CRP can play some functional roles instead of acting as an inflammation marker only (Pepys et al., 2006; Matsuda et al., 2011; Simons et al., 2014; Koenig, 2017; Jimenez et al., 2018; McFadyen et al., 2018; Molins et al., 2018; Sproston et al., 2018). Clinically, CRP is often found to be very useful to stratify the patients for treatment; for example, it was recently reported that the magnitude of CRP reduction following an anti-inflammatory therapy by canakinumab might provide a simple clinical method to identify individuals most likely to accrue the largest benefit from continued treatment, which further suggest that lower is better for inflammation reduction (Ridker et al., 2017, 2018). Furthermore, it is unlikely that our body would like to use its valuable resources to generate a large quantity of CRP as a non-functional waste. These accumulating evidences motivate us to experimentally examine the causality of CRP by directly observing the phenotypes and exploring the molecular mechanisms caused by its chronic low-grade elevation.

## Materials and Methods

### Creation of Human CRP Transgenic Rats

The transgene contained the human CRP gene (21-bp fragment before the transcription starting site, the exons and intron, and 1.2 kb of 3′-flanking region), and the mouse albumin promoter (from +22 to −300 bp) and enhancer (from −12.171 to −9.469 kb) (Supplementary Figure 1A). Purified DNA was microinjected into fertilized eggs obtained by mating CD IGS Sprague–Dawley rats from Charles River Laboratory (Wilmington, MA) at the University of Michigan Transgenic Animal Model Core Facility. Transgenic rats were identified by PCR with transgene-specific primers (Supplementary Figures 1B,C) using genomic DNA samples obtained from tail biopsies. Positive transgenic founder rats were bred with Sprague–Dawley (SD) rats to establish the transgenic lines. Animals were housed in the Center for Laboratory Animal Resources of Morehouse School of Medicine. Transgenic human CRP rats were given water *ad libitum* and a standard rat chow (Laboratory Rodent Diet 5001, LabDiet, USA).

### Human CRP Expression of in Transgenic Rats

The expression of the human CRP transgene and rat endogenous gene was measured by quantitative real-time PCR (qRTPCR) (Supplementary Figure 1D), ELISA (Supplementary Figure 1E) and Western blot analysis (Supplementary Figure 1F).

Total RNA samples were extracted from the liver with the RNeasy Mini Kits (Qiagen) after homogenization with FastPrep Lysing Matrix Tubes D (MP Biomedicals, Germany). Reverse transcription was performed with 0.5 μg of total RNA using a SuperScriptIII First-Strand Synthesis kit (Invitrogen). The real-time PCR was performed with a Faststart DNA Master SYBR Green kit (Roche) on a Light Cycler.

Whole blood was withdrawn from the tail vein of the animals. The serum was obtained by a centrifugation at 13,000 rpm at 4°C for 10 min. ELISA was performed to measure the protein levels of human CRP expression using a human CRP specific ELISA kit (HELICA Biosystems, Fullerton, CA) and rat CRP expression using a rat-specific ELISA kit (US Biological, Swampscott, MA). The serum samples were diluted 1:6,000 fold for the human CRP ELISA and 1:20,000 for the rat CRP ELISA, and the ELISA experiments were carried out following the manufacturers' protocols.

For the Western blot experiments, the serum was diluted at 1:1000, and then loaded onto 4–12% Bis-Tris polyacrylamide gels. It was transferred to PVDF membranes, blocked, and then incubated with anti-human CRP antibody (ab52687, Abcam, 1:500 dilution). Membranes were then incubated with the secondary antibody (goat anti-mouse IgG-HRP, Santa Cruz, Sc-2005, 1: 2,000 dilution), washed three times, and then incubated with substrate solution (SuperSignal West Pico Chemilluminescent Kit) for 5 min and exposed to X-ray film.

### Body Weight and Body Mass Index (BMI) Measurement

Longitudinal growth in all animals was followed up from the age of 5 to 40-weeks old. Body weight, and body length (nose-anus length) were measured weekly in the morning throughout the follow-up period. The body weight and body length were used to determine the body mass index as follows: BMI = body weight (g)/length^2^ (cm^2^).

### Measurement of Fat Mass by Magnetic Resonance Imaging (MRI)

MRI had become one of the ultimate methods to directly measure the fat distribution in animal models. In this study, we scanned the transgenic rats and the control rats on a 3T MRI scanner (MAGNETOM Trio, Siemens Healthcare, Germany) with the Siemens 4-channel flex coil in the Emory Yerkes National Primate Research Center primate center. The structural T1-weighted images of the whole body were acquired using a spin-echo sequence with the acquisition parameters: TR = 2,520 ms, TE = 15 ms, FOV = 340 × 340 mm, data matrix = 384 × 384, slice thickness = 2 mm, 50 slices, 6 averages. Total scan duration was 24 min for each rat. Raw data from the MRI scans were processed using the MRIcron software toolkit to covert to analytical format and create, view, and analyze the three-dimensional images. The quantitative measurements of fat in all rats were carried out on the sagittal slice crossing the renal hilum with the Image J software.

### Food Consumption

Food consumption was measured daily in the morning at the same time (9:00 to 10:00 am) continuously from 8 to 12-weeks old for 1 month. Every morning, sufficient food was added after being weighed. The next morning, the residual food was weighed again. The difference was the consumptive food by rats in the same cage. Based on food and caloric intake, the following nutritional parameter was calculated: Energy intake (kJ/day) = mean food consumption × dietary metabolic energy.

### Pathological Phenotyping

The tissues of fat and spleen were collected. The organs were weighted, and gross appearance was examined immediately. The collected tissues were kept in 10% buffered formalin, embedded, and cut into 5-micron-thicksections. The tissue sections were mounted on slides and stained with hematoxylin & eosin (H&E) or Oil Red O. The spleen sections were further stained with anti-CD3 antibody (Santa Cruz, sc-20047) to demonstrate the T lymphocytes, and with anti-CD20 antibody (Santa Cruz, sc-7735) to show the B lymphocytes. The secondary antibodies were sc-2039 for CD3 and sc-2042 for CD20. The images were taken by the Picture FrameTM Application 2.3. The number of splenic white pulps was counted at 4×magnification in 10 areas of the spleen sections from either transgenic rats or control rats. The results of Immunohistochemistry were quantified by Image-Pro Plus software (version 6.0). The pathological experiments were performed by the Histo-Pathology Core of Atlanta VA Medical Center, and pathological interpretations were performed by the Emory Department of Pathology.

### Lipid Profiling

The rats were fasted overnight for 15 h and blood was withdrawn from caudal vein. The prepared serum was either assayed immediately or stored in aliquots at −80°C for later use, avoiding repeated freeze/thaw cycles. The lipid profiles were measured by the Emory Lipid Research Laboratory of Atlanta Clinical and Translational Science Institute (ACTSI). Briefly, triglyceride (TG) was measured in serum by enzymatic method with the Triglyceride Quantification Kit (ab65336, abcam, MA, USA); total cholesterol (TC), high-density lipoprotein cholesterol (HDL-C), and low-density lipoprotein cholesterol (LDL-C) were measured with the HDL and LDL/VLDL Cholesterol Assay Elisa Kit (ab65390, abcam, MA, USA).

### Hematological Analysis

We carried out a complete blood count (CBC) to evaluate the composition and concentration of the cellular components of blood in the transgenic rats and control rats. Blood was withdrawn from the 26-week-old transgenic rats and the control rats (line 488, *n* = 5; line 519, *n* = 6; controls, *n* = 4). All blood samples were sent to Antech Diagnostics (Morrisville, North Carolina) for the hematological analysis. Siemens Advia 120 Multispecies Hematology System was used in the hematological analysis.

### Proteomic Examinations

Blood was withdrawn from 16-to 17-week-old rats, stored at 4°C overnight, and then centrifuged at 3,000 rpm at 4°C for 15 min. Highly abundant plasma proteins, such as albumin, IgG, and transferrin, were depleted from samples using a Multiple Affinity Removal Column (Agilent Technologies, Cat No. 5188-5218). The samples were then concentrated, and total protein concentrations were measured with a BCA Protein Assay Kit (Thermo Scientific, Cat No. 23225). About 300 μg of depleted serum proteins were dissolved in 200 mM triethyl ammonium bicarbonate (TEAB). Following reduction and alkylation, serum proteins were digested into peptides with trypsin (Promega, Cat No. PRV5111) at 37°C overnight. Peptides were then labeled with TMT6 (126 - 131 Da) (Thermo Scientific, Cat No. 90066). TMT126, 127, and 128 were used to label control (non-transgenic) samples in triplicates, and TMT129, 130, and 131 were used to label samples from the transgenic rats in triplicates. Labeled peptides were combined and fractionated with strong cation exchange (SCX) column (Thermo Scientific) in high-performance liquid chromatography (HPLC) (Shimazdu) and analyzed in an TQ-Orbitrap XL instrument (Thermo-Fisher Scientific) coupled to an Ultimate 3000 Dionex nanoflow LC system (Dionex, Sunnyvale, CA). Peptides were identified according to high mass resolution, and TMT6 reporter ion quantification was recognized with high energy collision dissociation (HCD). Proteins were searched for each identified peptide with Mascot (Matrix Science, Boston, MA). The ratios of TMT6 reporter ion abundances in MS/MS spectra generated by HCD (up to six reporter ions ranging from m/z 126.12 to m/z 131.14) were used to calculate fold changes of proteins between the control groups and the transgenic groups. From the raw proteomic results, all proteins with *Σ*#unique peptides <2 and *Σ*coverage <8 were removed from the final report. Only those proteins with ≥1.5-fold increase or decrease were included in the pathway analysis in Gene Ontology (GO) and PathCards via the GeneCards portal.

### Enzyme-Linked Immunosorbent Assay (ELISA)

ELISA was used to measure blood levels of a series of hormones using a series of specific commercial kits. ELISA experiments were outsourced to the ACTSI/Emory Cardiovascular Specialty Laboratories (Atlanta, Georgia). Briefly, serum samples were collected from transgenic lines (488 and 519) and their non-transgenic littermates (controls). Insulin and glucagon were measured by ELISA using the commercial kits. The sources of commercial ELISA kits are as follows: Insulin (Mercodia), Glucagon (R&D Systems), triiodothyronine (T3) (Cal Biotech), total thyroxine (T4) (Cal BioTech), thyroid-stimulating hormone (TSH) (ALPCO), Leptin (Crystal Chem), Adiponectin (R&D Systems), TNFα (R&D Systems), IL6 (R&D Systems), and Complement C3 (Kamiya Biomedical).

### High-Throughput Sequencing Analysis of Intestinal Microflora

Total microbial DNA was extracted from fecal samples using Qiagen stool DNA extraction kit (Cat. No. 51504) following the manufacturer's protocol and then sequenced by the 16S RNA sequencing on an Illumina HiSeq2500 sequencer. Sequences were grouped into operational taxonomic units (OTUs) using the clustering program UCLUST and assigned OTUs into taxonomic categories using the Ribosomal Database Silva_111 16S rRNA database. PCoA was calculated with the QIIME pipeline. The Ribosomal Database Program classifier was used to assign taxonomic category to all OTUs at confidence threshold of 0.97. The Ribosomal Database Program classifier uses Silva_111 16S rRNA database (http://www.arb-silva.de/), which has taxonomic categories predicted to the genus level.

### Statistical Analysis

All data were expressed as mean ± standard deviation; the statistical significance was analyzed by Student's *t*-test two-tailed for paired data or by one-way analysis of variance (ANOVA) using the Bonferroni *post-hoc* test for multiple comparisons. The linear regression was used to investigate the relationship between the body weight and the age of rats. In our experiment, the body weight was treated as the corresponding variable, and the age (week) was employed as the explanatory variable in the statistical analysis. Different datasets were combined based on the timeline (week is used as the time unit) of up to 40 weeks. After the combination, there were a total of three groups of rats, line 519, line 488, and non-transgenic littermates of SD rats included. The significance levels were set at 0.05 for all tests. The SAS statistical package 9.3 (SAS Institute, Inc., Cary, North Carolina) was used for all data managements and analyses.

## Results

### Chronic CRP Elevation Induces Adult-Onset Obesity

**Figure 1:**
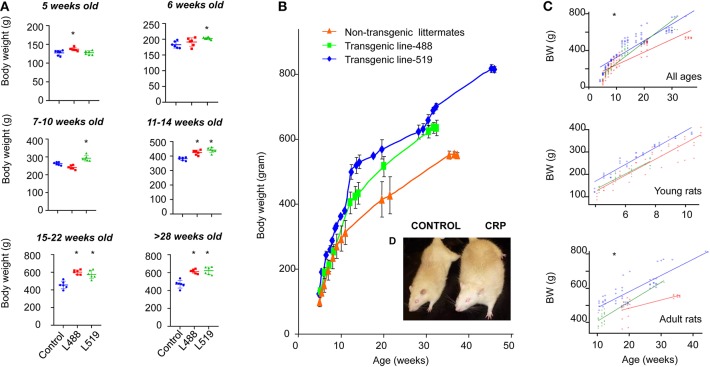
Adult-onset obesity observed in the CRP transgenic rats. **(A)** Body weight is shown by mean and SD at different ages. The *p*-values of student *t*-test are shown above the bar. Male rats of male human CRP transgenic line 519 (*n* = 6), line 488 (*n* = 6) and their non-transgenic littermates (*n* = 6) were included in this experiment (*p < 0.05). **(B)** The growth curve of the human CRP transgenic rats and their non-transgenic littermates. Means and standard deviations (SD) are showed at each time point for line-519 (male, *n* = 13), line-488 (male, *n* = 8) and the non-transgenic group (male, *n* = 9). Among the rats younger than 10-week old, body weight of transgenic rats is not substantially higher than the non-transgenic littermates; when rats are older than 11 weeks old, the body weight of transgenic rats start to be higher than the control rats. According to the results obtained from the univariate linear regression analysis of scatter plot of body weight using age as explanatory variable, growth rates are similar between transgenic rats and control rats before 10 weeks old [42.2 (transgenic line 488), 38.5 (transgenic line 519), and 37.6 (control group)]; however, when the rats are older than 11-weeks old, the growth rates of transgenic rats are about 2-fold higher than the control rats [11.8 (transgenic line 488), 9.5 (transgenic line 519), and 5.0 (the control rats)]. These results indicated that it is adult-onset obesity developed in these rats. **(C)** Univariate linear regression on the scatter plot of body weight using age as explanatory variable, young rats, ≤10 weeks old, adult rats, ≥11 weeks old, *p < 0.001; **(D)** Gross appearance of a male 1-year-old CRP transgenic rats and a male 1-year-old control rat.

We noticed that the CRP transgenic rats were big and strong, so we started to monitor the longitudinal growth of body weight and nose–anus length during the period from 5-weeks old to 40-weeks old. We found that the human CRP transgenic rats were larger in gross appearance, with higher body weight than their non-transgenic littermates (Figure 1 and Supplementary Figure 2). Univariate linear regression analysis of scatter plot of body weight using age as explanatory variable showed that, among the rats younger than 10-weeks old, the growth rates were 42.2 (transgenic line 488), 38.5 (transgenic line 519), and 37.6 (control group), but among the rats older than 11-weeks old (adulthood), the growth rates were 11.8 and 9.5 for two transgenic lines and 5.0 for the control rats (Figure 1C). These results showed that the long-term chronic CRP elevation could lead to overweight.

### Validation of Obesity by Whole-Body Magnetic Resonance Imaging (MRI)

**Figure 2:**
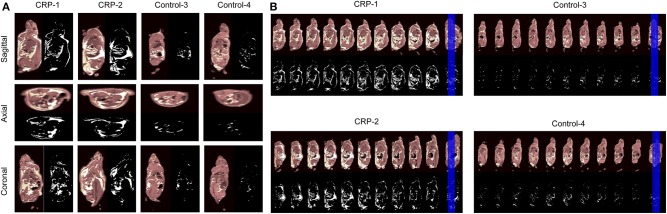
Body fat distribution accessed by MRI. In order to examine whether the higher body weight of CRP transgenic rats is due to increased fat volume, we measured the body fat distribution by MRI. Male 26-week-old rats of human CRP transgenic line 519 (*n* = 1), line 488 (*n* = 1) and their non-transgenic littermates (*n* = 2) were included in this experiment. **(A)** The views at the sagittal slices, the axial slices and the coronal slices. The fat volume is quantitated with the Image J software. The total fat volume of transgenic rats was 6-9 fold higher than control rats (Sagittal, 6.0-fold; Axial, 6.7-fold; Coronal, 8.9-fold). Among the fat mass, 11.8% was subcutaneous fat, and 88.2% was intra-abdominal fat in transgenic rats. About 63.0% of body fat was the omental fat; **(B)** Continuous sagittal sections scanned from nose-to-tail throughout the body. In these continuous sections, all of them are across the renal hilum. The pictures are shown in gray-scale and black-white, in which white areas indicate the fat. These MRI data confirmed that the CRP transgenic rats developed visceral obesity with accumulated intra-abdominal visceral fat.

The fat volume was measured by a standard T1-weighted (T1w) whole-body magnetic resonance imaging (MRI) to validate that these transgenic rats had more fat. As shown by the 2D images on the sagittal slices, the axial slices, the coronal slices (Figure 2A), the continuous sagittal scans from nose to tail (Figure 2B) and the 3D images (Supplementary Video 1), the fat volume was significantly higher in the CRP transgenic rats than the fat mass of control rats. The total fat mass of transgenic rats was 6- to 9-fold higher than the fat mass of control rats (Sagittal, 6.0-fold; Axial, 6.7-fold; Coronal, 8.9-fold). Among the total body fat in the transgenic rats, 12–23% was subcutaneous fat, 77–88% was intra-abdominal fat, and 42–63% was the omental fat. These MRI results clearly showed that the observed higher body weight caused by long-term chronic CRP elevation was due to high fat volume, validating the existence of obesity in these animals.

### Chronic CRP Elevation Induces Hypertrophic Obesity

**Figure 3:**
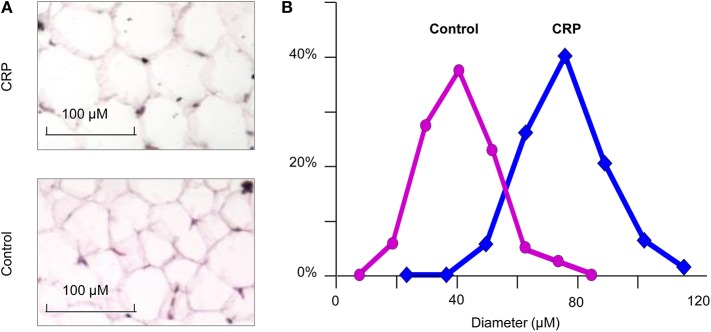
The size of adipocytes in the CRP and control rats. **(A)** Histochemical staining of fat tissue (*n* = 6, 16-week-old, male); **(B)** Comparison of transgenic rats and control rats on the size of adipocytes. *p = 4.5E-30.

We stained the cells collected from visceral fat by HE and measured their cell sizes. The diameter of adipocytes in the CRP rats (75.8 ± 13.4 microns) was significantly (*p* = 3.1E-48) higher than the control rats (51.7 ± 10.9) (Figure 3). The adipocytes of the CRP rats were about three times bigger than the control rats. These data indicated the presence of adipocyte hypertrophy in these rats.

### Excessive Food Intake Is Not Required for CRP-Induced Obesity

Food intake was followed up among rats from 8 to 12-weeks old. We found no significant difference between transgenic rats and control rats (Supplementary Figure 3). We also measured leptin and adiponectin, two adipokines that modulated satiety and energy expenditure. The results showed no differences on the levels of adiponectin and leptin between transgenic rats and control rats.

### Chronic CRP Elevation Induces the Activation of Innate Immune System

**Figure 4:**
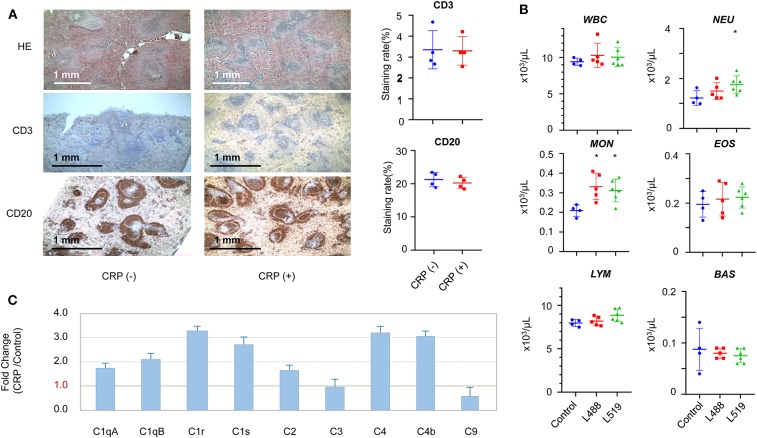
The changes in the immune system induced by chronic CRP elevation. **(A)** Splenic remodeling. Staining by HE, anti-CD3 (T-cells), and anti-CD20 (B-cells) antibodies (4×), higher density of white pulps was observed in the transgenic rats compared with controls (*p* = 1.8E-6). Male 26-week-old rats of human CRP transgenic line 519 (*n* = 2), line 488 (*n* = 2) and their non-transgenic littermates (*n* = 4) were included in this experiment. **(B)** The hematological results of total WBC (white blood cells), neutrophils (NEU), lymphocytes (LYM), monocytes (MON), eosinophils (EOS), basophil (BAS), and absolute large unstained cells (LUC) are shown. Male 26-week-old rats of human CRP transgenic line 519 (*n* = 6), line 488 (*n* = 5) and their non-transgenic littermates (*n* = 4) were included in this experiment (*p < 0.05). **(C)** The subcomponents of the complement system. Male 16 to 17-week-old rats of human CRP transgenic line 519 (*n* = 4), line 488 (*n* = 4) and their non-transgenic littermates (*n* = 8) were included in this experiment.

CRP transgenic rats displayed a noteworthy splenic remodeling. The number of white pulps (the reservoir of T-cells and B-cells) in the CRP transgenic rats was about 2.5-fold higher than control rats (*p* < 0.001) (Figure 4A). The staining with anti-CD3 and anti-CD20 antibodies showed that the cell contents in each of these white pulps were similar between transgenic rats and control rats, suggesting that the CRP transgenic rats had more T-cells and B-cells than control rats in their spleens (Figure 4A).

In concert with the splenic remodeling, we observed striking differences in the white blood cell profiles between the CRP transgenic rats and control rats. Systemic hematological examinations showed that the counts of total WBC, neutrophils, lymphocytes, monocytes and eosinophils were higher in transgenic rats than those of control rats, and the basophil count was lower in transgenic rats than control rats (Figure 4B). The differences on the monocyte count reached statistical significance (*p* < 0.05) between transgenic rats and control rats, and the difference on the neutrophil count reached statistical significance only in one transgenic line (Figure 4B). Moreover, these CRP rats showed significantly lower RBC (red blood cells) counts (*p* < 0.05) (Supplementary Figure 4).

In concert with the splenic remodeling, we also observed striking differences in the subcomponent levels of complete system between the CRP transgenic rats and control rats. The subcomponents of complement system in the classical pathway, including C1qA, C1qB, C1r, C1s, C2, C4b, and C4, were all upregulated by 2-to 4-fold, and the membrane attack subcomponent C9 was down-regulated (Supplementary Table 1). Complement C3 was not changed (Figure 4C).

### Chronic CRP Elevation Induces Changes in Factors Involved in Energy Expenditure

Our results showed remarkable differences between the CRP transgenic rats and control rats on the protein levels on a series of the key proteins that play critical roles in the control of immune response and energy expenditure. Corticosteroid-binding globulin (CBG, also called transcortin or Serpina6) was substantially upregulated by 2.2-fold in the CRP rats compared with the control rats (Supplementary Table 1). Two other proteins, RBP4 (Retinol Binding Protein 4) and CAP1 (Adenylyl Cyclase-Associated Protein 1) were remarkably down-regulated in transgenic rats (RBP4, 0.58-fold; CAP1, 0.44-fold) (Supplementary Table 1). Attractin (Atrn, 0.49-fold), Inter-alpha-trypsin inhibitor heavy chain H4 (ITIH4, 2.1-fold), Fetuin-B (Fetub, 0.27-fold), Alpha-1-acid glycoprotein (also called Orosomucoid, Orm1, 1.6-fold), tropomyosin alpha-4 chain (Tpm4, 0.56-fold), haptoglobin (Hp, 0.33-fold), and serum paraoxonase/arylesterase 1 (Pon1, 1.68-fold) (Supplementary Table 1) were also remarkably affected by the chronic elevation of the human CRP in the transgenic rats.

We measured the serum levels of T3 and its prohormone, T4, as well as TSH produced from the pituitary gland (Supplementary Figure 5). Those CRP transgenic rats had significantly higher T4 levels (line 488, 4.75 ± 0.54 μg/dl, *p* = 0.028; line 519, 4.72 ± 0.34 μg/dl, *p* = 0.024) than the control rats (3.87 ± 0.64 μg/dl). Their T3 levels (line 488, 0.59 ± 0.08 ng/ml; line 519, 0.57 ± 0.09 ng/ml) seemed to be slightly lower than those of the control rats (0.63 ± 0.08 ng/ml) but did not reach statistical significance. On the plasma concentration of TSH, there was no statistical significance between transgenic line 488 and the control rats, but transgenic line 519 had significantly lower TSH compared with the control rats (line 488, 0.80 ± 0.38 ng/ml; line 519, 0.26 ± 0.17 ng/ml; control, 0.89 ± 0.84 ng/ml).

### Chronic CRP Elevation Induces the Changes of Apolipoproteins and Lipoproteins

The blood concentrations of a series of apolipoproteins (ApoA1, ApoA2, ApoA4, ApoC1, ApoC2, ApoC4, ApoE, and ApoM) were about 2-fold higher in the CRP rats than the levels in the control rats; the concentration of apolipoprotein ApoB-100 was decreased by 1.6-fold (Supplementary Table 1, Supplementary Figure 6). The triglyceride levels in the transgenic rats (line 519, 1.69 ± 0.78 nmol/μl; line-488, 0.72 ± 0.07 nmol/μl) were statistically significantly higher than the control rats (0.45 ± 0.17 nmol/μl) (*P* = 0.003 for line-519 vs. control; 0.014 for line-488 vs. control) (Supplementary Figure 7). We observed no difference on TC, HDL, and LDL between transgenic rats and control rats under the normal diet.

### Gut Flora Associated With Obesity Phenotypes Caused by CRP Elevation

**Figure 5:**
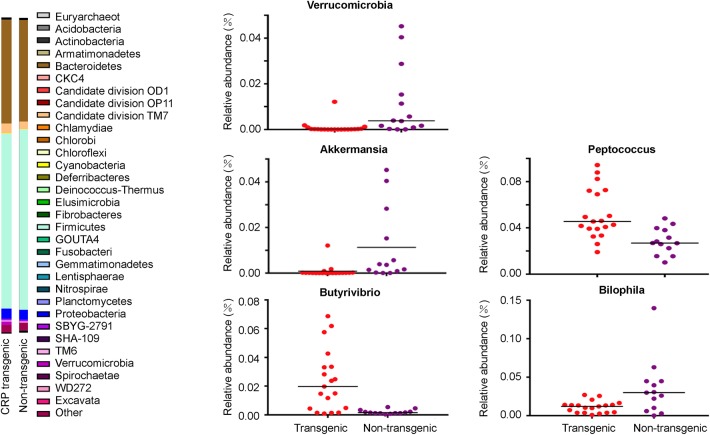
Gut flora in the CRP rats. Intestinal microflora was examined by 16S sequencing. Phylum-level proportional abundances are shown on the left. Relative abundance of Verrucomicrobia Richness represented as the proportions of OTUs classified at the phylum rank; relative abundance of *Akkermansia, Bilophila, Butyrivibrio*, and *Peptococcus* Richness represented as the proportions of OTUs classified at the Genus rank. Each dot represents one biological sample; there are 32 biological samples in total, including 19 male CRP rats and 13 male control rats at 16 weeks old.

Our sequencing results showed that Firmicutes and Bacteroidetes were the two predominant phyla among the bacterial groups, contributing 57.7 and 32.5% of gut flora in the transgenic rats, and 55.5 and 32.9% in the non-transgenic littermates. Proteobacteria and Actinobacteria constituted the next dominant phyla at 3.2 and 0.3% in transgenic rats, and 3.3 and 0.4% in the non-transgenic littermates (Figure 5). The abundance of Verrucomicrobia in the transgenic rats was significantly lower (*p* < 0.05) than the non-transgenic group (Figure 5). At the genus level, *Lactobacillus, Sphingomonas, Blautia, Candidatus, Saccharimonas, Bacillus, Bacteroides, Desulfovibrio, Sphingomonas, Alloprevotella*, and *Akkermansia* were the abundant genera (>1%).

Among the dominant genera, the abundance of *Akkermansia* was significantly lower (*p* < 0.05) in the transgenic rats compared with the non-transgenic littermates (Figure 5, Table 1). Among the other genera, the abundance of *Butyrivibrio* (11.6-fold) and *Peptococcus* (1.8-fold) were significantly higher (*p* < 0.05, Table 1) in the transgenic rats than in the non-transgenic rats (Figure 5, Table 1), the abundance of *Bilophila* in the transgenic rats was 3-fold lower (*p* < 0.05) than that in non-transgenic littermates (Figure 5, Table 1). These results clearly showed that CRP induced remarkable changes in gut flora composition, including the well-known “weight-loss bacteria” (Bradlow, 2014) *Akkermansia muciniphila*.

**Table 1:** Relative abundance (%) of gut flora at the genus level.

**Bacteria**	**CRP**	**Non-CRP**	**Ratio**
Butyrivibrio^**^	0.237 ± 0.214	0.020 ± 0.015	11.571
Sphingomonas	1.02 ± 3.44	0.85 ± 1.68	1.203
Ruminococcus	2.96 ± 1.00	2.80 ± 1.30	1.059
Prevotella	7.36 ± 5.10	6.17 ± 6.74	1.191
Lactococcus	0.52 ± 0.27	1.11 ± 1.18	0.465
Lactobacillus	11.648 ± 5.66	9.32 ± 6.55	1.25
Desulfovibrio	1.09 ± 0.55	1.368 ± 0.96	0.795
Candidatus Saccharimonas	2.432 ± 0.86	3.049 ± 2.28	0.798
Blautia	9.302 ± 2.68	10.424 ± 6.36	0.892
Bacteroides	1.524 ± 0.92	1.087 ± 1.34	1.403
Bacillus	2.496 ± 1.32	4.526 ± 5.46	0.552
Alloprevotella	0.646 ± 0.50	1.118 ± 1.64	0.578
Akkermansia^*^	0.085 ± 0.28	1.128 ± 1.16	0.076
Bilophila^*^	0.011 ± 0.008	0.035 ± 0.037	0.322
Peptococcus^**^	0.05 ± 0.02	0.03 ± 0.01	1.808

* *Means with different superscripts differed significantly (p < 0.05)*.

** *Means with different superscripts differed significantly (p < 0.01)*.

### Global Changes of the Protein Profiles Induced by Chronic Elevation of CRP

In total, 52 proteins displayed a ≥ 1.5-fold difference (1.5-fold higher or 0.66-fold lower) on the serum levels between transgenic rats and non-transgenic littermates (Supplementary Table 1). Among these proteins, 20 proteins showed a ≥2-fold higher concentrations than the controls, and 10 proteins showed a ≤0.5-fold lower concentrations than the controls. Among these 52 proteins, 7 were uncharacterized proteins or rat-specific proteins (Supplementary Table 1). Bioinformatic pathway analysis showed that these proteins mainly hit several systems, including the complement components of the innate immune system (as reported above in the section of immune system), apolipoproteins (as reported above in the section of lipid metabolism), coagulation system (as reported above in the section of coagulation system), energy homeostasis system and vasodilation (Supplementary Tables 2–5).

## Discussion

This study demonstrates that the long-term chronic CRP elevation will lead to adult-onset obesity, which is based on results of the CRP transgenic rat model with low levels of human CRP expression. As shown by whole-body MRI scanning, fat mass of transgenic rats is 6-to 9-fold higher than the fat mass of control rats, among which 12–23% is subcutaneous fat, 77–88% is intra-abdominal fat, and 42–63% is omental fat. The adipocytes in the CRP rats (mean diameter = 75.8 microns) is three-times bigger than the fat cells in the control rats (mean diameter = 51.7 micron). The increase of adiposity might be an essential factor in expression of CRP and chronic inflammation leading to obesity, which needs to be further explored.

CRP has been known as an inflammation marker, but our results show that CRP is more than a marker; it can play an active role to provoke systematically the innate system. Chronic CRP elevation regulates the levels of complement components in the classical pathway (Figure 4C); it modulates the blood count of neutrophils, lymphocytes, monocytes, eosinophils, and basophils (Figure 4B), and it also remarkably remodels the spleen, the largest lymphoid organ, in which the number of white pulps (reservoirs of T-lymphocytes and B-lymphocytes) increased by about 2.5-fold (Figure 4A). In fact, CRP belongs to the Pentraxin family, a highly conserved protein family that has profound effects on the regulation of the innate immune system (Du Clos, 2013; Cox et al., 2014; Simons et al., 2014; McFadyen et al., 2018); it may participate in recognizing pathogens, activating the complement system and interacting with Fcgamma receptors (Du Clos and Mold, 2004; Peisajovich et al., 2008). It was discovered recently that the progressive process of obesity is accompanied by chronic inflammation; even modest weight gain was associated with inflammatory activation (Christ et al., 2018; Piening et al., 2018), and the obesity caused by western diet is accompanied by augmented innate immune system (Thorburn et al., 2014). It is possible that chronic low-grade elevation of CRP may pass on a “cheating” message of non-communicable inflammation to the body, which then over-reacts to this cheating message and develops obesity.

Besides the innate immune system, CRP also remarkably modulates the key proteins in the regulation of energy expenditure and immune response (Supplementary Table 1). Among these proteins, CBG (or called transcortin, 2.2-fold) is a key regulator of liberating fuel, maintaining cardiovascular homeostasis, consolidating memories of a stressful event (Reul and Chandramohan, 2007; Roozendaal et al., 2009), and acting as a “brake” on the innate immune response to promote the resolution of inflammation (Walker, 2007). Cortisol is a well-known stress hormone; a strict control (GC) of glucocorticoid hormone responses to stress is essential for health. Most (GCs) in blood are bound to CBG; only unbound hormone molecules can be released into target tissues to exert the physiological actions. The free fraction of circulating GCs constitutes only 3–5% of the total GC pool; CBG is the major factor influencing interindividual genetic variability of plasma GC levels in both basal and stress conditions. Therefore, the level of CBG determines the cortisol bioavailability in target tissues to exert their physiological actions (Henley and Lightman, 2011). Cortisol has been shown to induce obesity, insulin resistance, hypertension, glucose intolerance, dyslipidemia, depression, memory loss, impaired wound healing, osteoporosis, myopathy, and many other features (Fernandez-Real et al., 2002; Schafer et al., 2013). RBP4 (Retinol-binding protein, 0.58-fold), is a well-known adipokine that serves as a signal from fat to other organs to control fuel usage in response to changes in adipose tissue mass and energy status (Yang et al., 2005), CAP1 (Adenylyl cyclase-associated protein 1, 0.44-fold) is a functional receptor for human resistin (Lee et al., 2014), a molecular link between inflammation, insulin resistance, obesity, and other cardiometabolic diseases (Lazar, 2007). Attractin (Atrn, also called mahogany, 0.49-fold) is a low-affinity receptor for Agouti protein (He et al., 2001), its membrane-bound isoform is a receptor involved in controlling obesity and its secreted isoform is involved in the initial immune cell clustering during inflammatory responses by regulating the chemotactic activity of chemokines (Duke-Cohan et al., 1998). Others include ITIH4 (2.1-fold), Pon1 (1.68-fold), Orm1 (1.6-fold), Fetuin-B (0.27-fold), haptoglobin (0.33-fold), and Tpm4 (0.56-fold). These proteins are all stress hormones, they regulate the basal metabolic rate and innate immune responses as well as inflammatory activities. These results suggest that CRP may deliver a message to these stress hormones, and then initiate a series of systematic adaptations (such as body weight and immune system) to prepare for the upcoming potential stresses.

*Akkermansia muciniphila* is a well-known “weight-loss bacteria” (Everard et al., 2013; Plovier et al., 2017). Now growing evidence suggests that the gut flora contributes to host metabolism and obesity (Cani and Delzenne, 2011; Le Chatelier et al., 2013). Low-grade inflammation is recently linked to gut flora composition (Schroedl et al., 2014). and low-grade inflammation associated with weight gain is at least partially due to the microbiome (Cani et al., 2008). In concordance to these recent findings, we found that CRP elevation could re-shape the composition of the gut flora, especially the weight-reducing bacteria, *A. muciniphila* (Figure 5). Thinking about the signaling passage from low-grade inflammation—gut flora—obesity, it is likely that CRP is upstream to the low-grade inflammation by bringing in a pseudo inflammatory signal for the low-grade inflammation.

In human, CRP is consistently the strongest factor associated with overweight and obesity as revealed by large epidemiological studies (Timpson et al., 2011). Many environmental factors have been known to elicit the chronic elevation of CRP independently of genetic factors. These environmental insults include infections, tissue injury, pollutions (Peters et al., 2001), social economic status (SES) (Nazmi and Victora, 2007; Brummett et al., 2013), dietary content (Fung et al., 2001; King et al., 2003) and pattern (Lopez-Garcia et al., 2004), stress, unhealthy lifestyles, and physical activities (Ford, 2002). These factors can driver chronic non-communicable inflammations (Mega et al., 2015). Interestingly, all of these factors are known to be closely related to the development of obesity. These factors may cause obesity through the “CRP—chronic non-communicable inflammation signal—innate immune system” pathway.

## Conclusion

In summary, long-term CRP elevation can lead to adult-onset obesity. CRP can also provoke a broad adjustment in the innate immune system and energy expenditure system. CRP may be a new therapeutic target for obesity and its complications.

## Data Availability Statement

The raw data supporting the conclusions of this article will be made available by the authors, without undue reservation, to any qualified researcher.

## Ethics Statement

All animal experiments were performed with the approval of the Animal Care Committee of Morehouse School of Medicine, conformed to the Guide for the Care and Use of Laboratory Animals published by the National Institutes of Health.

## Author Contributions

Conceptualization: QS. Data curation: YC, SY, YD, QiW, and QL. Formal analysis: XZ, WL, and YM. Investigation: QL, QingwW, WX, YM, QiW, QingW, WL, XZ, SY, JZ, GW, and LZ. Methodology: QL, DE, MB, JC, and LM. Resources: QingwW, QS, QL, and BW. Supervision: QS and QL. Writing-original draft: QL, QiW, YM, WX, and QingwW. Writing-review and editing: QS.

### Conflict of Interest

The authors declare that the research was conducted in the absence of any commercial or financial relationships that could be construed as a potential conflict of interest.

## Supplementary Material

Supplementary Figure 1 The creation of a CRP transgenic rat model.

Supplementary Figure 2 Visualization of CRP transgenic rats and non-transgenic littermates.

Supplementary Figure 3 Food intake and energy intake.

Supplementary Figure 4 The RBC traits.

Supplementary Figure 5 Thyroid hormones.

Supplementary Figure 6 Apolipoproteins in the CRP rats.

Supplementary Figure 7 Lipoprotein profiles.

Supplementary Video 1 Three-dimensional MRI view of the transgenic rats and the control rats.

Supplementary Table 1 Proteomic profiling.

Supplementary Table 2 Protein-associated biological processes (Gene Ontology).

Supplementary Table 3 Summary of biological processes (Gene Ontology) associated with the CRP elevation.

Supplementary Table 4 Protein-associated biological processes (PathCards).

Supplementary Table 5 Summary of SuperPath (PathCards) associated with the CRP elevation.
