# Supplementary material for: C-Reactive Protein Causes Adult-Onset Obesity Through Chronic Inflammatory Mechanism
Source: Front Cell Dev Biol. 2020 Feb 20;8:18. doi: 10.3389/fcell.2020.00018 (PMC7044181; doi:10.3389/fcell.2020.00018)
Supplement: Supplementary Figure 1 — The creation of a CRP transgenic rat model. [file Data_Sheet_1.PDF]

Supplemental Figures

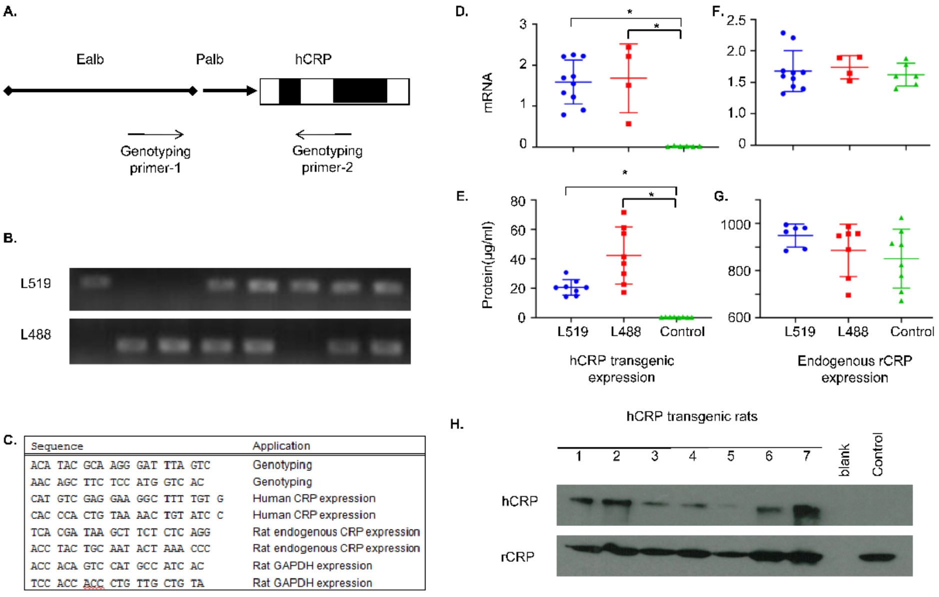

Sequence	Application
ACA TAC GCA AGG GAT TTA GTC	Genotyping
AAC AGC TTC TCC ATG GTC AC	Genotyping
CAT GTC GAG GAA GGC TTT TGT G	Human CRP expression
CAC CCA CTG TAA AAC TGT ATC C	Human CRP expression
TCA CGA TAA GCT TCT CTC AGG	Rat endogenous CRP expression
ACC TAC TGC AAT ACT AAA CCC	Rat endogenous CRP expression
ACC ACA GTC CAT GCC ATC AC	Rat GAPDH expression
TCC ACC ACC CTG TTG CTG TA	Rat GAPDH expression

Figure S1. The creation of a CRP transgenic rat model. A. The transgene structure. The transgene is composed of a mouse albumin enhancer, a mouse albumin promoter, and human CRP gene. B. Genotyping results by PCR of some rats including transgenic rats and non-transgenic littermates. The positions of genotyping PCR primers are shown under the transgene structure. C. The sequences of the primers. D and F. The mRNA expression of human CRP transgene or rat endogenous CRP in the liver measured by quantitative real-time PCR (qRTPCR) and normalized by GAPDH mRNA levels. Male 16-week-old rats of human CRP transgenic line 519 (n=10), line 488 (n=4) and their non-transgenic littermates (n=6) were included in this experiment. E and G. The protein concentration of human CRP or rat endogenous CRP in the blood measured by high-sensitivity ELISA. Male 16-week-old rats of human CRP transgenic line 519 (n=8), line 488 (n=8) and their non-transgenic littermates (n=8) were included in this experiment. (*$p<0.05$) H. Western blot analysis confirmed the existence of the pentameric human CRP and dimeric rat CRP in transgenic rats (3 rats in line-519 and 4 rats in line-488); a blank lane and a non-transgenic littermate was included. (d) The mRNA expression of human CRP transgene in the liver measured by quantitative real-time PCR (qRTPCR) and normalized by GAPDH mRNA levels. L519, transgenic line-519; L488, transgenic line-488; control, gender and age matched non-transgenic littermates. hCRP, human CRP; rCRP, rat endogenous CRP.

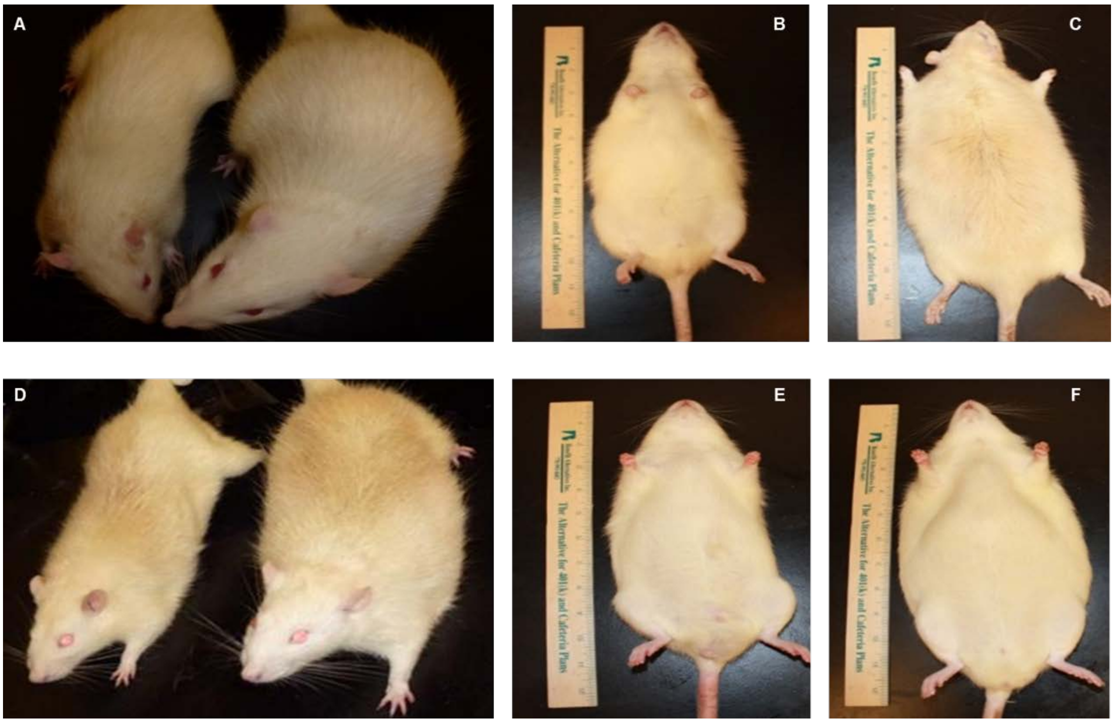

Figure S2. Visualization of CRP transgenic rats and non-transgenic littermates. One male transgenic rat (line-519) and one male control rat are shown in this figure. A-C. 17-week old, D-F. 1-year old. The non-transgenic SD rats were on the left, and the transgenic rats were on the right. B&E. non-transgenic SD rats; C&F. transgenic rats.

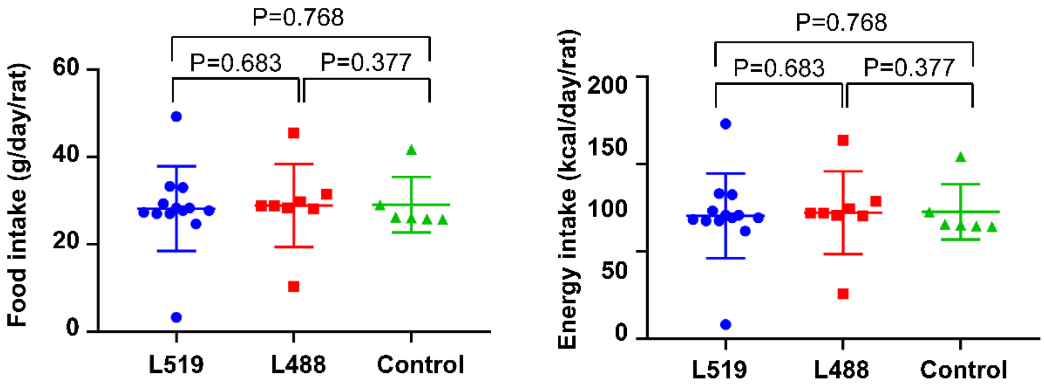

Figure S3. Food intake and energy intake. Male 8 to 12-week-old rats of human CRP transgenic line 519 (n=13), line 488 (n=8) and their non-transgenic littermates (n=6) were included in this experiment. Values are shown as mean and standard deviation. There was no significant difference observed between these 3 groups.

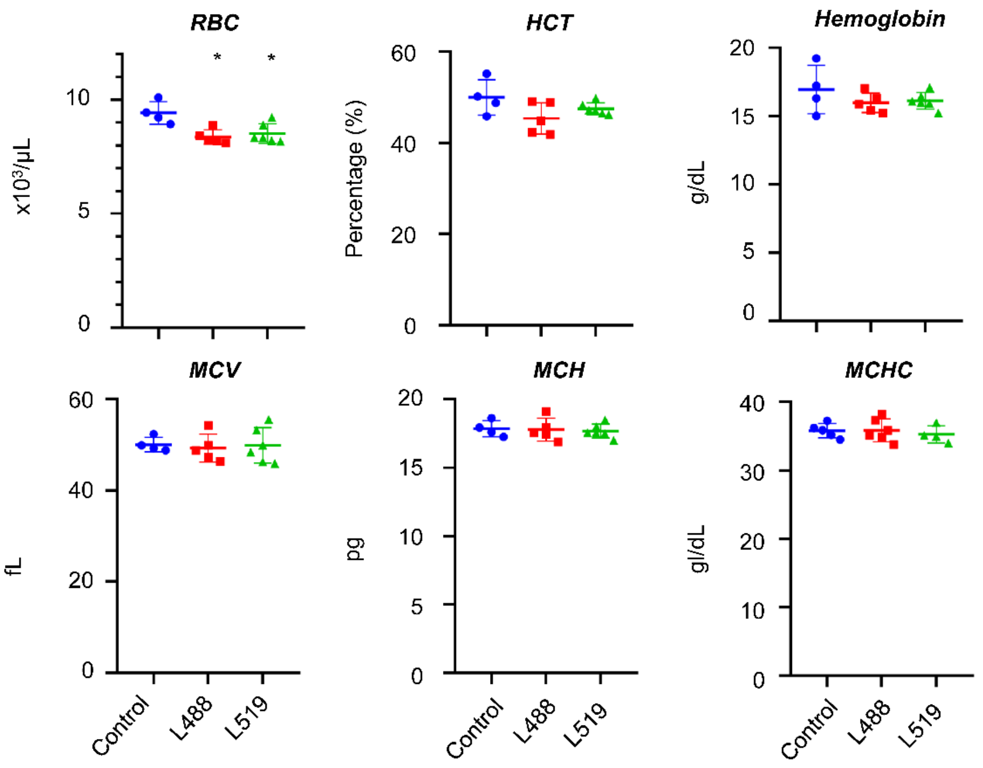

Figure S4. The RBC traits. Male 26-week-old rats of human CRP transgenic line 519 (n=6), line 488 (n=5) and their non-transgenic littermates (n=4) were included in this experiment. RBC (red blood cells) count of transgenic rats is lower than control rats. Hematocrit (HCT) of transgenic rats seems to be lower than the HCT of control rats but did not reach the statistical significance. No difference was observed on other RBC indices in the standard CBC lab tests, including hemoglobin, mean corpuscular hemoglobin (MCH), mean corpuscular hemoglobin concentration (MCHC), mean corpuscular volume (MCV) and hemoglobin (HB). These results show that the CRP transgenic rats have less red blood cells, but normal hemoglobin and normal cell size(*$p<0.05$).

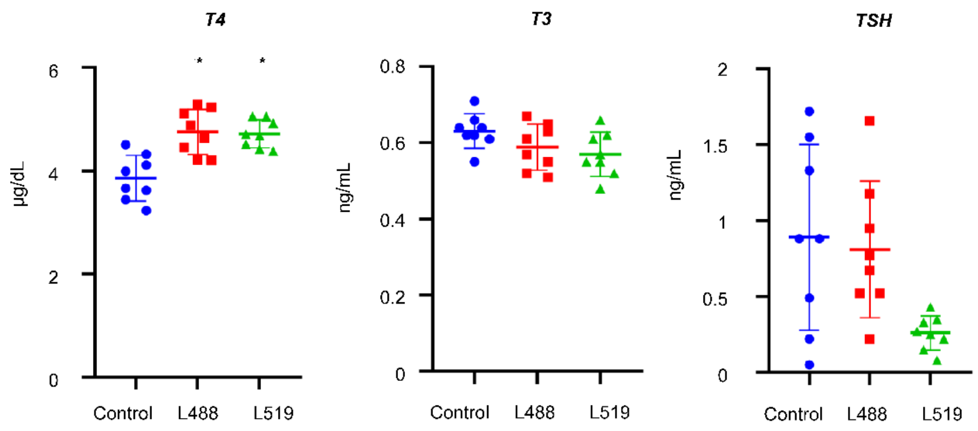

Figure S5. Thyroid hormones. Male 26-week-old rats of human CRP transgenic line 519 (n=8), line 488 (n=8) and their non-transgenic littermates (n=8) were included in this experiment. In our study, we found that these CRP transgenic rats have significantly higher T4 levels than the control rats. Their T3 levels seemed to be slightly lower than the control rats but did not reach the statistical significance. There is no statistical significance on the plasma concentration of thyroid-stimulating hormone (TSH) between transgenic line-488 and the control rats, but transgenic line-519 has significantly lower TSH compared with the control rats(*$p<0.05$).

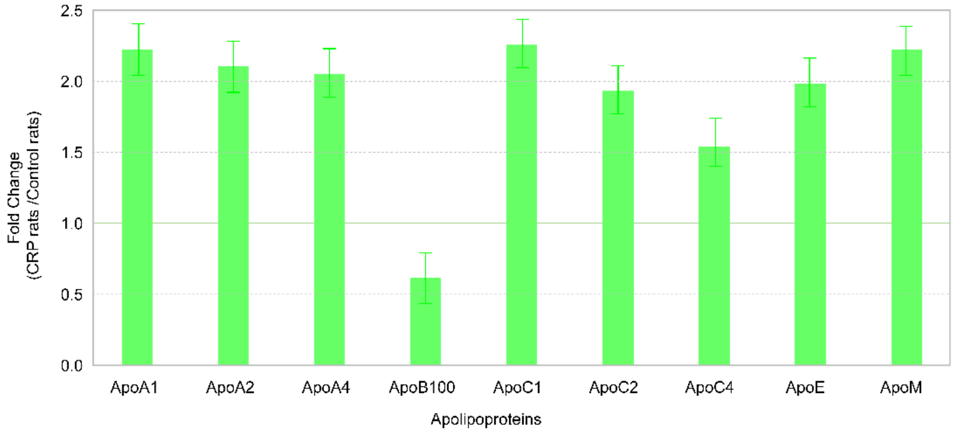

Figure S6. Apolipoproteins in the CRP rats. Male 16 to 17-week-old rats of human CRP transgenic line 519 (n=4), line 488 (n=4) and their non-transgenic littermates (n=8) were included in this experiment. Our results showed that the chronic elevation of CRP increases the production of the apolipoproteins of ApoA1, ApoA2, ApoA4, ApoC1, ApoC2, ApoC4, ApoE, and ApoM, and decreases the production of ApoB-100, indicating that the body under long-term CRP elevation has received a message that it needs less ApoB and but need more of the other apolipoproteins(*$p<0.05$).

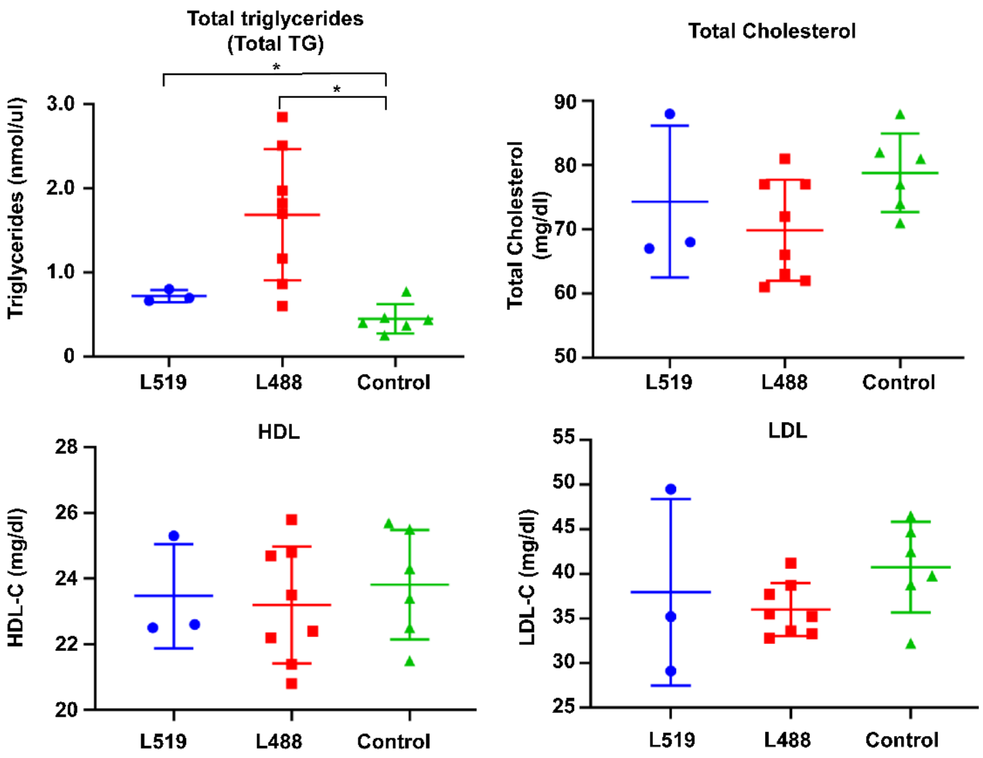

Figure S7. Lipoprotein profiles. Male 16 to 17-week-old rats of human CRP transgenic line 519 (n=3), line 488 (n=8) and their non-transgenic littermates (n=6) were included in this experiment. Each lipoprotein particle contains apolipoproteins, triglyceride (TG), cholesterol, phospholipids. The triglyceride levels in the transgenic rats were statistically significantly higher than the control rats. No difference was observed on total cholesterol, HDL and LDL between the CRP rats and control rats under the normal diet(*$p<0.05$).
